# Silicon Mediated Plant Immunity against Nematodes: Summarizing the Underline Defence Mechanisms in Plant Nematodes Interaction

**DOI:** 10.3390/ijms232214026

**Published:** 2022-11-14

**Authors:** Jingwen Yu, Xiyue Yu, Caihong Li, Muhammad Ayaz, Sulaiman Abdulsalam, Deliang Peng, Rende Qi, Huan Peng, Lingan Kong, Jianping Jia, Wenkun Huang

**Affiliations:** 1 State Key Laboratory for Biology of Plant Diseases and Insect Pests, Institute of Plant Protection, Chinese Academy of Agricultural Sciences, Beijing 100193, China; 2 Cotton Sciences Research Institute of Hunan, Changde 415101, China; 3 Institute of Plant Protection and Agro-Products Safety, Anhui Academy of Agricultural Sciences, Hefei 230041, China; 4 Department of Crop Protection, Division of Agricultural Colleges, Ahmadu Bello University, Zaria 810106, Nigeria

**Keywords:** silicon, plant-nematode interaction, interaction mechanism, defence response, nematode management

## Abstract

Silicon (Si) is known to stimulate plant resistance against different phytopathogens, i.e., bacteria, fungi, and nematodes. It is an efficient plant growth regulator under various biotic and abiotic stresses. Silicon-containing compounds, including silicon dioxide, SiO_2_ nanoparticles (NPs), nano-chelated silicon fertilizer (NCSF), sodium siliconate, and sodium metasilicate, are effective in damaging various nematodes that reduce their reproduction, galling, and disease severity. The defence mechanisms in plant-nematodes interaction may involve a physical barrier, plant defence-associated enzyme activity, synthesis of antimicrobial compounds, and transcriptional regulation of defence-related genes. In the current review, we focused on silicon and its compounds in controlling plant nematodes and regulating different defence mechanisms involved in plant-nematodes interaction. Furthermore, the review aims to evaluate the potential role of Si application in improving plant resistance against nematodes and highlight its need for efficient plant-nematodes disease management.

## 1. Introduction

Silicon (Si) is the second most abundant element in the earth’s crust, and its importance in agriculture has increased multifold [1]. Si is known to enhance growth and stimulate plant-induced resistance against nematodes. Plant-induced resistance is a physiological state of enhanced defensive capacity elicited by environmental stimuli, including fungi, bacteria, viruses, nematodes, and insect herbivores [2]. Based on the elicitor’s nature and the regulatory pathways involved, the two most clearly defined forms of induced resistance are systemic acquired resistance (SAR) and induced systemic resistance (ISR) [3]. ISR is enhanced by plant growth-promoting rhizobacteria without accumulating pathogenesis-related proteins [4]. Unlike ISR, SAR can be triggered by exposing the plant to pathogenic microbes or artificially with chemicals such as salicylic acid (SA), thiamine, and inorganic salt, coupled with the accumulation of pathogenesis-related proteins (e.g., chitinase, glucanase, etc.) and hypersensitive response. In general, neither chemical elicitor exhibits any direct antimicrobial activity. However, after treatment with chemical elicitors, the levels of defence-related genes in the SA pathway were upregulated in plants that express SAR resistance, while the levels of defence-related genes in the methyl jasmonate (JA)/exogenous ethylene (ET) pathway were upregulated in plants to express ISR resistance. The combination of the two pathways enables a broader spectrum of plant resistance to pathogens [4,5,6]. Nahar et al. found that exogenous application of ET and JA on the shoots induced a strong systemic defence response in the rice roots against root-knot nematode (RKN) *Meloidogyne graminicola* and ET-induced defence requires an intact JA pathway, while JA-induced defence was still functional when ET signalling was impaired [4,5,6]. However, Lohar and Bird did not observe significant changes in susceptibility plants *Lotus japonicus* with ET resistance, but foliar application with JA was shown to induce systemic defence of tomatoes against RKN [7,8].

The application of Si is beneficial to plant growth and production. Plant species greatly differ in silicon accumulation due to differences in root uptake capacity [9]. Numerous studies show that silicon accumulation positively affects many crops (e.g., rice, sugarcane, corn). Silicon improves plants’ mechanical and physiological properties and helps plants to overcome various abiotic and biotic stresses [10,11,12]. For example, by stimulating defence reactions, Si enhances host resistance to various pathogenic fungi, bacteria, and nematodes [13,14,15]. Chérif et al. observed that soluble silicon activated defence in cucumber against *Pythium* spp. through enhanced activity of chitinases (CHT), peroxidases (POD), and polyphenol oxidases (PPO), and increased the accumulation of phenolic compounds [16]. Rodrigues et al. reported that Si-mediated resistance to *Magnaporthe grisea* in rice was associated with a higher accumulation of antimicrobial compounds at infection sites [17]. Berry et al. observed that the total numbers of plant-parasitic nematodes and *Pratylenchus zeae* and *Helicotylenchus dihystera* in the soil were significantly lower in plots where foliar Si levels were higher [18]. Guimarães et al. showed that the reduction of *M. incognita* in potassium silicate-treated sugarcane plants was associated with the enhancement of POD activity, while the reduction of root-knot nematodes (RKN) in horse bean and coffee plants treated with calcium silicate was associated with the production of lignin, PPO or phenylalanine ammonia-lyase (PAL) [19,20]. However, the mechanistic basis and regulation of Si-mediated resistance against most plant-parasitic nematodes (PPNs) are still poorly understood in higher plants. 

There are over 4000 species of PPNs, and they represent an important constraint on global agricultural production [21]. Damage caused by PPNs has been estimated at $US 80 billion per year [22]. Numerous studies have reported that silicon plays an important role in plant-nematode interactions and increases plant resistance to nematode diseases. Recent studies showed that several silicon nanoparticles with nematicidal activity were used against RKNs [23,24]. Many Si-containing compounds are eco-friendly and environmentally safe, promising results in managing PPNs. Silicon-mediated resistance of plants against nematode and related physical, biochemical and molecular interaction mechanisms have been demonstrated in Table 1, which will be discussed in detail in the following sections.

## 2. Role of Silicon in Nematode Management

Nematode diseases are the major concern in agricultural production, resulting in a drastic reduction in crop yield and quality. Silicon compounds, including silicon dioxide, SiO_2_ nanoparticles (NPs), nano-chelated silicon fertilizer (NCSF), sodium siliconate, and sodium metasilicate, have been used to control different PPNs. Junior et al. observed that root irrigation with SiO_2_ at a dose of 0.6 g dm^−3^ of soil negatively affected the formation of galls of *M. incognita* in the inoculated tomatoes and reduced the final population of nematodes in the root system [25]. However, Ardakani et al. revealed that SiO_2_ NPs (11–14 nm) at concentrations of 200~800 mg mL^−1^ were found not to affect the mortality of *M. incognita* in tomatoes [26]. Foliar spray of SiO_2_ also caused a higher reduction in root galling and multiplication of *M. incognita* in carrots [27]. The nematode population in carrots was decreased by 40%, and the number of galls was decreased by 30% in the application of 0.10 mg mL^−1^ SiO_2_ NPs compared to the control. In addition, Khan et al. revealed that foliar spray of 0.20 mg mL^−1^ SiO_2_-NPs in plants reduced the juveniles of *M. incognita* and improved up to 37.92% of dry shoot weight and increased 70.42% of chlorophyll content in eggplant [28]. Besides the positive effect on reducing nematode number, SiO_2_ NPs also showed a great inhibitory effect on egg hatching of nematodes. Danielle and Claudia found that the hatching of *M. javanica* eggs was significantly reduced in silicon [29]. Ahamad and Siddiqui also observed that SiO_2_ NPs at 0.05 and 0.10 mg ml^−1^ concentrations caused 69.91% and 91.67% inhibition of hatching of *M. incognita*, respectively, over control after 48 h [27]. An in vitro test also showed that Si NPs concentrations significantly inhibited the percentage of egg hatching at a different time of exposure than in the control. The mortality rate of juveniles ranged from 87% to 98% with 100 and 200 ppm Si NPs after 72 h [30]. In other in vitro experiments, Si was also found to have a positive effect on reducing the *M. paranaensis* population [31]. Dugui-Es et al. observed that root application of sodium metasilicate at the rate of 400 ppm gave the lowest number of eggmasses in cucumber, and application of Si at 200 ppm both on the leaves and roots significantly reduced the number of galls in inoculated plants [32].

To date, dozens of reports have documented Si’s capacity for improving the resistance of vegetables and other economically important crops to different nematodes. Khan and Siddiqui reported that seed priming and foliar spray of SiO_2_ NPs caused a reduction in root galling, nematode multiplication, and disease indices of *M. incognita* in beetroot. Seed priming with SiO_2_ NPs at 200 mg L^−1^ resulted in the lowest galling and nematode multiplication, significantly different from that in a foliar spray [33]. Following the foliar application of silicon nanoparticle solution to tomatoes, Udalova et al. observed that silicon was accumulated in the parasitic zone, activating the stress response mechanisms to *M*. *incognita* infection and inhibiting nematode proliferation [34]. Furthermore, Zhan et al. reported that amendment with silicon fertilizer reduced nematodes’ number of *M. graminicola* in rice roots and delayed their development [35]. At a dose of 0.04%, amendment of Si resulted in a significant reduction of nematodes (53.1%) and root galls (65.5%) at 14 dpi, and the ratio of adult females in 0.04% Si-treated plants (73%) was significantly lower than that of non-treated plants (92%).

Further research disclosed that increased resistance in rice was correlated with higher transcript levels of defense-related genes in the ethylene (ET) pathway. In addition, Al-Banna et al. observed that silica nanoparticles caused the degeneration of the reproductive organs of nematodes, and dead nematodes were found to exhibit black internal organs [23]. These observations indicated that the application of Si increased the resistance of plants to defend themselves against different nematodes.

In many countries, solid fertilizers of Si are integrated into the soil, while liquid irrigation is used for foliar application or soil amendment. Si fertilizer’s liquid and solid forms increased plant resistance to different nematodes. In a two-year’s field experiment, Sinh et al. observed that the abundances of PPNs, especially *Hirschmanniella* spp., were significantly reduced after treatment with silicate fertilizer in acid sulfate soil [36]. In a greenhouse experiment, Charehgani et al. indicated that soil drenching of NCSF significantly reduced the nematode population indices [37]. The reproduction factor of nematode in pre- and post-treated tomatoes with NCSF as soil drenches at the rate of 1000 mg per plant was reduced by 66% and 44%, respectively, compared to the control. In the case of silicon dioxide nanoparticles (nSiO_2_), soil drenches at 1000 mg per plant nSiO_2_ reduced the reproduction factor by 50% and 27%, respectively. Mansourabad et al. found that the application of sodium siliconate (Na_2_O_3_Si) in combination with iron sequestrene (Fe-EDDHA) on *M. incognita* infected cucumber significantly reduced the number of root galls by 55% compared to control after 60 days [38]. The supply of Si effectively enhanced Si accumulation in cotton plants, and a lower population of *M. incognita* was estimated in Si fertigation [39]. The nematode population in cadusafos (Cad) treatment (0.08 mL of 1.6 mg L^−1^ cadusafos) did not differ from Cad + Si treatment (0.04 mL of 1.6 mg L^−1^ cadusafos +500 mL of 2 mmol L^−1^ potassium silicate), as both treatments resulted in lower nematode population at 180 dai. The combination between SiNPs and half-recommended doses (RD) of nematicides reduced nematode reproduction, gall formation, egg masses on eggplant roots, and the final population of juveniles in the soil. Applying 200 ppm Si NPs + 0.5 RD fenamiphos resulted in a significant reduction of galls by 79.1% and eggmasses by 81.5% than the control, which is similar to that of RD of fenamiphos. Similar results were also observed in the treatments of 200 ppm SiNPs + 0.5 RD fosthiazate [30]. Thus, fertilizers of silicon can be used to control different nematodes with root irrigation or soil application.

## 3. Physical Mechanisms of Silicon-Mediated Nematode Resistance

Plant-parasitic nematodes display a wide variety of interactions with their hosts. Some nematodes are migratory ectoparasites that never enter the host, but some endoparasites use their styles to penetrate plant cells and cause extensive damage [40]. Successful infection of pathogens requires entering the host plant by penetrating physical barriers, including wax, cuticles, and cell walls [41]. At the same time, silicon accumulation can improve the overall mechanical strength of plants and form a cuticle-Si double layer to prevent pathogen penetration, thereby decreasing disease incidence [42]. To facilitate penetration, nematode juveniles often use a combination of physical damage through thrusting the stylet and breakdown of the cell wall by cellulolytic and pectolytic enzymes. In *Radopholus similis* infected plants, several cell wall-degrading enzymes, including cellulases, xylanases, and pectate lyases, were identified, and they help nematodes to penetrate banana plants [40]. In *Ditylenchus dipsaci*-infected potatoes, nematodes were also found to release enzymes that soften cell walls and facilitate feeding on the parenchymatous cells of the cortex. In a pot experiment, Silva et al. observed that the penetration of *M. exigua* on the roots of coffee plants was negatively affected by Si treatment [43]. The number of *M. exigua* juveniles inside the roots was reduced by 25% at 5 dai and by 77% at 10 dai in +Si plants than that of −Si plants.

Lignin and phenolic secondary metabolism play important roles in PPN resistance [44]. To reduce the adverse effects of nematodes, phenolic compounds were often produced in cell walls or epidermal tissues to enhance plants’ resistance to nematodes. A higher deposition of callose and accumulation of phenolic compounds were observed at 24- and 48-h post-inoculation (hpi) in Si-amended inoculated plants than in nonamended inoculated plants [35]. Adding calcium silicate to Si-deficient soil significantly increased the concentration of lignin thioglycolic acid derivatives of coffee. It decreased the number of galls and eggs of *M. exigua* [45]. Dutra et al. observed that the amendment of plants with calcium silicate increased the secondary lignin metabolism of roots and remarkably reduced the number of root galls and eggs of various species of *Meloidogyne* in beans, tomatoes, and coffee [20]. In addition, Haegeman et al. found that *Bursaphelenchus xylophilus* possesses unique plant cell-wall-modifying proteins, glycoside hydrolase family (GHF) 45 cellulases, which help *B. xylophilus* overcome the obstacle of the pinewood cell wall with a distinct parasitism mode from other PPNs [46]. For future studies, the availability of a genome sequence may present an excellent opportunity to analyze nematode parasitism and plant responses to a non-biotrophic pathogen.

**Table 1:** **Effect of silicon on plant nematode management and related interaction mechanisms**.

Hosts	Nematodes	Compounds	Interaction Mechanisms	Reference
Beetroot	*Meloidogyne incognita*	Silicon dioxide	Increase the activities of SOD, CAT, PPO and POL; reduce root galls and nematode multiplication	[33]
Carrot	*M. incognita*	Silicon dioxide	Reduce root galling and nematode multiplication, inhibit egg hatching	[27]
Cucumber	*M. incognita*	Sodium metasilicate	Reduce eggmasses and root galling	[32]
Cucumber	*M. incognita*	Sodium siliconate	Reduce root galls	[38]
Coffee	*M. exigua*	Calcium silicate	Increase PPO and PAL activity; decrease root galls and eggs	[45]
Coffee	*M. exigua*	Calcium silicate	Reduce juveniles	[43]
Horsebean	*M. incognita*	Calcium silicate	Increase production of lignin PPO and PAL, reduce root galls and eggs	[20]
Cotton	*M. incognita*	Potassium silicate	Decrease nematode population	[39]
Eggplant	*M. incognita*	Silicon nanoparticles	Maximize nematicidal efficiency; inhibit egg hatching	[30]
Eggplant	*M. incognita*	Silicon nanoparticles	Reduced the juveniles and increased shoot dry weight of plant	[28]
Rice	*M. graminicola*	Silicon	Generate ROS of rice, activate ET pathway, reducenematode number and delay its development	[35]
Sugarcane	*M. incognita*	Potassium silicate	Increase POD activity of plant	[19]
Tomato	*M. incognita*	Silicon carbide nanoparticles	Degenerate reproductive organs of nematode	[23]
Tomato	*M. incognita*	Silicon carbide nanoparticles	No effect on nematode mortality	[26]
Tomato	*M. javanica*	Nano-chelated silicon fertilizer	Reduce the nematode population indice	[37]
Tomato	*M. incognita*	Silicon dioxide	Reduce the final population of nematodes	[25]
Tomato	*M. incognita*	Silicon Nanoparticles	Accumulate silicon in the parasitic zone	[34]
Petri dish	*M. javanica*	Silicon	Reduce egg hatching	[29]

## 4. Biochemical and Molecular Mechanisms of Silicon-Mediated Nematode Resistance

Si increases plant biochemical resistance through enhanced activity of defence-associated enzymes (e.g., PPO, POD, PAL, glucanase), production of antimicrobial compounds, e.g., flavonoids, phenolics, pathogenesis-related (PR) proteins, and phytoalexins. Si also induces biochemical resistance by regulating systemic signals via phytohormones, e.g., ET, SA, and JA [47,48]. Si-enhanced resistance increases the activity of many defence-related enzymes during plant-pathogen interaction. Several studies have shown that activities of defence-related enzymes were activated after treatment with Si, including chitinase (CHT), PPO, POD, PAL, b-1,3-glucanase, superoxide dismutase, ascorbate peroxidase, glutathione reductase, catalase, lipoxygenase, and glucanase, then increased the resistance of plants [49]. PPO is the main enzyme of phenolic substance oxidation, and its activity has been positively correlated with plant disease resistance [50]. Cucumber plants infected with powdery mildew fungus supplied with Si showed PPO and PAL-enhanced activity compared to untreated plants [51]. In Si treatments 10 days after inoculation, PPO and PAL activities increased by 54.9% and 26.6%, respectively, and the number of galls and eggs significantly decreased in *M. exigua*-infected coffee plants [45].

Lignin is covalently linked to hemicellulose and crosslinks different plant polysaccharides conferring mechanical strength to the cell wall against many lytic enzymes that pathogens produce during host tissue colonization [52]. A higher concentration of lignin-thioglycolic acid (LTGA) derivatives was observed on the roots of coffee plants supplied with Si and was directly associated with the reduction of RKN reproduction [45]. Silicon absorbed by plants can be quickly transferred to the roots and produce defense-related substances by activating the synthesis of phenols, lignin, and callose, thus defending against the infection of nematodes. Guimarães et al. found that the activity of POD was significantly increased in potassium silicate-treated sugarcane plants, and the number of *M. incognita* was significantly reduced than that of untreated plants [19]. Dutra et al. (2004) observed that calcium silicate application decreased the number of root-knot nematodes in horsebean and coffee plants. Si-mediated resistance in these plants was associated with the production of lignin, POD, PPO, or PAL [20]. Khan and Siddiqui found that the application of SiO_2_ NPs as seed priming and foliar spray to beetroots inoculated with *M. incognita* plus *Pectobacterium betavasculorum* resulted in a significant increase in the activities of defence enzymes, including SOD, CAT, PPO, and PAL, supporting that Si induced resistance also inhibited the development of RKN nematode [33].

Two types of resistance mechanisms (pre-infection and post-infection resistance) for RKN have been reported in cowpea, peanut, and cucumber [53]. For pre-infection resistance, nematodes cannot enter plant roots due to toxic or antagonistic chemicals in the root tissue. For post-infection resistance, nematodes can enter plant roots but fail to develop into male or female nematodes due to an early immune response of the host plants. Additionally, Zhan et al. investigated whether Si can generate reactive oxygen species (ROS) in the rice-*M. graminicola* interaction [35]. The expression level of *OsRbohB,* an H_2_O_2_ synthesis gene involved in the plant immune response, showed the highest expression level in Si-treated inoculated treatment at all tested time intervals. Significant differences in H_2_O_2_ levels were observed at 6 hpi and 24 hpi after Si treatment compared with control roots. Si-treated plants showed induced H_2_O_2_ accumulation compared with non-treated plants by 140.8% at 6 hpi and 116.7% at 24 hpi. These data suggest that Si amendment may activate the rapid generation of ROS to induce defence against the infection of root-knot nematodes. In addition, a higher level of lignin was observed at 24, and 48 hpi in Si-amended inoculated plants than in no amended inoculated plants, indicating that cell wall lignification can be enhanced by Si amendment. The investigation of nematode behaviour and development showed that amendment with 0.04% Si significantly reduced nematodes at 14 dpi. The ratio of adult females in Si-treated plants was also significantly lower than that of non-treated plants. In contrast, a higher ratio of third-and fourth-stage juveniles was observed in Si-treated roots compared to non-treated roots.

To prevent pathogens infection, host plants have developed complicated immune systems, which are regulated by complex networks of signal transduction pathways, including JA, SA, and ET pathways [54]. Several studies have suggested that Si may regulate plant immune responses by modulating signalling pathways and phytohormone levels. Plant phytohormones were found to accumulate in Si-treated Arabidopsis plants, and the biosynthesis of SA, JA, and ET in leaves was stimulated to increase the resistance of the powdery mildew pathogen *Erysiphe cichoracearum* [55]. In tomato plants, the expression of the *ACCO* gene, which was involved in ethylene biosynthesis, was upregulated by Si when exposed to *Ralstonia solanacearum*, supporting that Si-induced resistance was mediated via ET signalling pathways [14]. Ethylene is involved in mediating plant responses to various biotic and abiotic stresses. Zhan et al. analysed the expression levels of genes involved in ET biosynthesis (*OsACS1*, *OsACO7*), ET signalling (*OsEIN2*), and ET response (*OsERF70*, *OsERF1*, *OsEBP89*) at different time intervals [35]. Transcriptions of the *OsERF1* and *OsEIN2* were significantly up-regulated in Si-amended plants at 24 hpi, and a significant enhancement of *OsACS1* expression was observed in Si-treated plants at 72 hpi.

Further investigation on the role of the ET response in two transgenic plants, *OsEIL1-2-RNAi*, which causes deficiencies in ET signalling, and *OsEIL1-OX*, which increases ET signalling by 15- to 20-fold, indicated that more nematodes were observed in the *OsEIL1-2-RNAi* plants than in the wild-type plants at 14 dpi. In contrast, significantly fewer nematodes were observed in the transgenic line *OsEIL1-OX* than in the wild-type plants. Fewer nematodes were observed in *OsEIL1-OX* transgenic plants after Si treatment. Therefore, the priming effect of silicon was only observed in the overexpressed transgenic lines but not in the insensitive lines. These results indicate that the ET signalling pathway is involved in rice’s Si-induced defence against *M. graminicola*.

## 5. Conclusions and Perspectives

**Figure 1:**
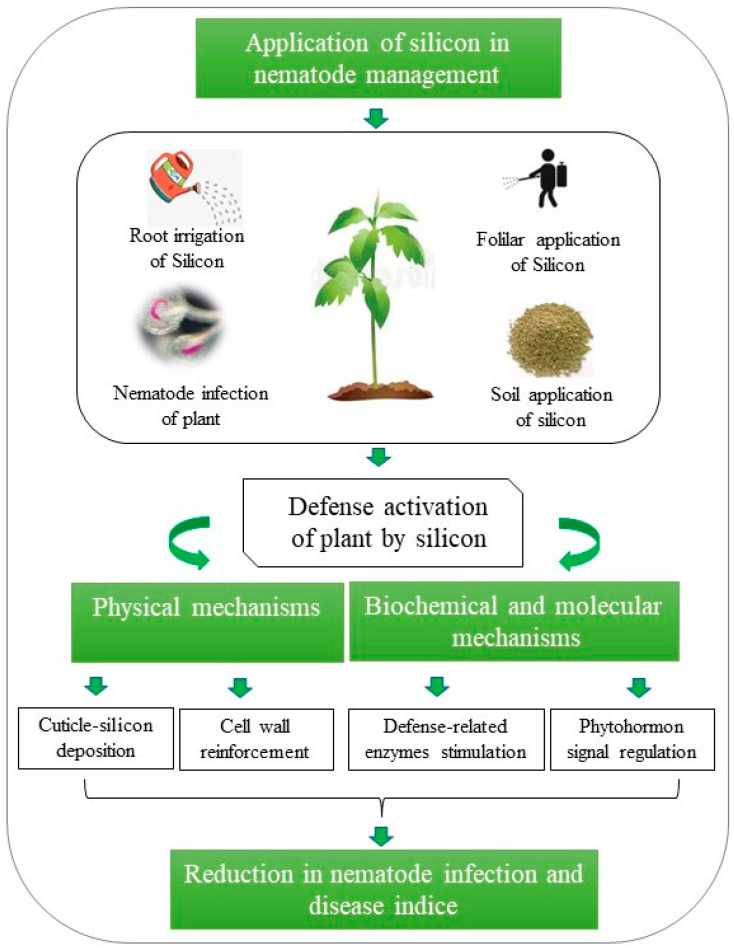
**Silicon mediated plant defense against nematodes and the interaction mechanisms of plant-nematodes.** Briefly, Si-containing compounds were used on plant roots or leaves to control a wide range of nematodes. In response to nematode attack on the plants, various defense mechanisms are activated by Si application. These defense mechanisms may involve physical, biochemical, and molecular defense. Physical mechanism embeds deposition of Si below the cuticle or reinforcement of cellwall, which hinders the entry of nematodes. Biochemical defense may involve stimulation of defense related enzymes that reduces the damage of nematodes. Molecular defense modulates defense-related genes that improves plant resistance against nematodes.

Plants will absorb silicon in the form of Si(OH)_4_ from soil or nutrient solutions, and silicon can be beneficial in protecting plants against biotic and abiotic stresses. Si is not classified as an essential plant nutrient (i.e., a component of metalloenzyme or macromolecules, or participating in electrochemical fluxes across membranes, such as potassium) or not yet an obvious component of signalling cascades (such as calcium). The current review presented the major role of Si in reducing nematode infection, galling, and disease severity. In trying to define the role of Si, various aspects of plant-nematodes interactions, i.e., physical, biochemical and molecular mechanisms, were investigated thoroughly. The available information related to plant defence mechanisms against nematodes has been summarized in Figure 1. Firstly, Si induces resistance against many nematodes by acting as a pre-formed defence barrier before nematode puncturation. Previous studies have shown that the reinforcement of plant resistance to nematode infection might be attributed to silicon accumulation in epidermal tissue [56]. Silicon absorbed in plants is known to form a binary film at the epidermal cell wall, which acts as a strengthening material to prevent nematode infections [57,58]. After treatment with Si, a thick layer beneath the cuticle was formed, and then the cell wall became less susceptible to enzymatic degradation by the nematodes [59].

Additionally, silicon may be deposited in the subcuticular layer and intercellular spaces [60]. This alters the anatomy and increases the silicification and accumulation of lignin and phenolic compounds in the lesions, enhancing the physical barrier and preventing nematodes’ penetration [61]. Secondly, the presence of silicon in a plant can improve the activity of plant defence enzymes and the regulation of JA, SA, and ET pathways. For example, PAL catalyses the phenylpropanoid pathway, resulting in the biosynthesis of the precursors of lignin [62]. The plant’s defence via lignification is a conserved basal mechanism in the plant’s immune response against pathogens [63,64]. Therefore, the increase in the activity of defence-related enzymes could prevent pathogenic infection in plants due to silicon supplementation. Finally, Si may regulate the expression of defence-related genes and effectors at a molecular level. As presented by Zhan et al., ethylene is involved in mediating rice responses to *M. graminicola* and those genes upregulated in ET signalling and ET response resulted in a significant reduction of nematodes [35]. Pathogen Effectors can modify host cell structure, metabolism and function and interfere with the triggering of host resistance [54]. The importance of effector proteins in a compatible host-pathogen interaction has been highlighted in the last few years [65,66]. Giraldo and Valent found that the haustorium of powdery mildew releases effectors into the cytoplasm to alter plant defences [54]. However, the reliance of nematodes on effectors to maintain their virulence and the site of Si deposition coinciding with effector release remains to be explored.

Although the benefits of Si fertilization on unstressed plants remain contentious, the same cannot be said for the expanding evidence supporting the positive role of Si in stressed plants [67]. The initial theory concerning the action mode of Si in plant prophylaxis involved the establishment of a mechanical barrier offering protection against the powdery mildew of cucumber [51]. However, Okuda & Takahashi measured leaf toughness and found that while Si protected the rice plant against blast disease, no observations have directly linked the fungus’s cell wall reinforcement with penetration failure [68]. Most studies hypotheses that soluble Si can act as a secondary messenger, a modulator of defence responses, have never been fully tested in the presence of a proper genetic model [12]. Vivancos et al. observed that, in the form of Si (OH)_4_, Si did not replace SA as a surrogate secondary messenger in the induction of defence reactions, which strongly suggests that other factors may play a role in the Si-mediated protection of plants against pathogens [69]. Furthermore, the control effect of most Si compounds against plant nematodes was not as well as that of chemical nematicides, suggesting that Si should be included as part of an integrated management program with a combination of pesticides and other biological agents.

Understanding the plant-nematode interaction mechanisms regulated by Si will help increase crop yield and enhance plant resistance against nematodes. Although several studies have explored the physical, biochemical, and molecular mechanisms of Si-mediated resistances, more research is required to uncover the phytohormone signal transduction and transcriptomic regulations involved in plant defence against nematodes [70]. Similarly, little is known about how Si transporters respond and how the Si transport pathway works under nematode attack [11]. In addition, limited data regarding the combined application of Si, fertilizers, and other biological agents to manage nematodes are available. Researchers should focus on long-term field studies rather than short-term greenhouse experiments to understand the unique defence mechanisms of Si. The application of Si compounds should not be the sole strategy in nematode management. Combining Si with other nematicides or biological agents will help improve the control effect of nematodes.

## Data Availability

Not applicable.
